# OA09 Vertebral fractures on bisphosphonates

**DOI:** 10.1093/rap/rkad070.009

**Published:** 2023-09-27

**Authors:** William Walshe, Anupama Nandagudi

**Affiliations:** Basildon University Hospital NHS Trust, Basildon, United Kingdom; Basildon University Hospital NHS Trust, Basildon, United Kingdom

## Abstract

**Introduction:**

Osteoporosis is a progressive skeletal disorder characterised by a decrease in bone mass and deterioration of bone tissue, leading to increased fragility and susceptibility to fractures. Risk factors include age, gender, post-menopause, genetics, hormonal imbalances, inadequate nutrition, sedentary lifestyle, and certain medications. Diagnosis involves bone mineral density (BMD) testing and assessment of fracture risk, using FRAX score. This patient presented with a fall, following a stroke, and multiple vertebral fractures while on bisphosphonates. She had an NG tube in situ which limited the treatment options. These fractures occurred despite adherence to the appropriate treatment guidance.

**Case description:**

A sixty-seven year old female was admitted to Basildon University Hospital following a fall due to a suspected stroke. A dedicated CT Trauma series reported no acute cervical spine fracture, but multiple compression fractures, more specifically of T9, T11, T12, L2, L4 and L5. As per radiology report, these were thought to be semi-recent, with no significant retropulsion and it was felt highly likely that these fractures were osteoporotic in nature. Further investigations confirmed that the patient had suffered a stroke (large acute infarct in the right MCA territory), and as a result had an unsafe swallow, requiring NG tube for feeding. This restricted the treatment options for the patient, especially regarding oral bisphosphonates, which would have been the initial preferred modality of treatment (and as shown below, had already been tried).

Regarding her prior medical history, she was previously under the care of rheumatology for the treatment of polymyalgia rheumatica which involved a prolonged course of glucocorticoid therapy, over a period of approximately two years. However, she was treated at another rheumatology department, and there was no available medical documentation to determine the entire course/pathway of this (and whether her response was complicated by flares), but this had been reduced to: prednisolone 6mg once daily in recent months. She has not been reviewed by rheumatology team for at least two years.

She was diagnosed with sero-positive rheumatoid arthritis (RA) in 1997, which had been well controlled on hydroxychloroquine 200mg once daily, although she had previously required: methotrexate (peak dosage 12.5mg once weekly, stopped in October 2020) sulfasalazine (peak dosage 1g once daily, stopped in December 2016).

**Discussion:**

While there were no DEXA scan results available, her myeloma screen was normal. As illustrated by her FRAX score below, she clearly presented an imminent fracture risk and was already being treated for osteoporosis with oral bisphosphonates at the time of her fall and vertebral fractures.

FRAX score (without DEXA), 10 year probability of fracture:

Major osteoporotic: 21%

Hip fracture: 5.1%

Her treatment at the time of the fall/fractures: alendronic acid 70mg once weekly and Calci-D 1000mg/1,000unit tablets once daily), since 2018. She had previously been treated with: risedronate 35mg (2015 to 2017) following diagnosis as being at risk of osteoporosis in 2014, at the age of fifty-eight. This was discontinued at the patients request, although no further reason was documented. She was also treated with hormone replacement therapy (climagest 1mg once daily) from 2008 to 2012.

This was a complex case considering previous treatments used (see above) and the current clinical status of the patient following her stroke (bedbound and intolerant of any oral medications). Luckily, osteoporosis treatment options extend beyond oral medications such as IV zoledronic acid, SC teraparatide or romosozumab, IV denosumab, etc.

Following a thorough assessment of the patients history and current clinical status, teriparatide was deemed the most appropriate treatment, primarily due to the presence of vertebral fractures, to promote bone healing. Teriparatide works as a synthetic form of PTH, stimulating bone formation and increasing bone density.

NOGG recommends: “Teriparatide or romosozumab as first-line treatment options in postmenopausal women at very high fracture risk, particularly in those with vertebral fractures”

NICE recommends: “In postmenopausal women with at least one severe or two moderate low-trauma vertebral fractures, teriparatide or romosozumab are recommended over oral bisphosphonates.”

**Key learning points:**

Despite treatment with multiple oral bisphosphonates over several years, this patient still suffered multiple vertebral fractures. Due to her past medical history, which included prolonged glucocorticoid therapy, being a post-menopausal female and a background of multiple chronic diseases (including RA), she was obviously at imminent fracture risk. Her treatment has only become more complicated following her stroke and inability to tolerate oral medication. This demonstrates the added difficulty in treating osteoporosis following vertebral fractures in a complex patient, and how challenging it can be due to several factors:

Firstly, vertebral fractures are usually indicative of advanced bone loss and compromised bone strength, making it more difficult to restore bone density and structural integrity.

Secondly, it is a chronic condition characterized by systemic bone loss, which means that treatment needs to address the underlying bone health throughout the body, not just the specific fracture site. This requires comprehensive management strategies to promote bone formation and prevent further bone deterioration.

Thirdly, vertebral fractures can be associated with pain and functional limitations, which can impact a patient's ability to engage in physical activities necessary for osteoporosis management, such as weight-bearing exercises.

Pain management becomes crucial to improve mobility and facilitate rehabilitation. This is further complicated following her stroke, where her mobility will be extremely limited.

Treating osteoporosis often involves a multifaceted approach that includes lifestyle modifications, such as adequate nutrition, regular exercise, and fall prevention strategies, alongside pharmacological interventions. Coordinating these various aspects of treatment and ensuring patient compliance can be complex, especially in a patient who has just suffered an extensive stroke.

